# Transitions between discrete and rhythmic primitives in a unimanual task

**DOI:** 10.3389/fncom.2013.00090

**Published:** 2013-07-22

**Authors:** Dagmar Sternad, Hamal Marino, Steven K. Charles, Marcos Duarte, Laura Dipietro, Neville Hogan

**Affiliations:** ^1^Departments of Biology, Electrical and Computer Engineering and Physics, Center for Interdisciplinary Research on Complex Systems, Northeastern UniversityBoston, MA, USA; ^2^Research Center “E. Piaggio”, University of PisaPisa, Italy; ^3^Department of Mechanical Engineering and Neuroscience Center, Brigham Young UniversityProvo, UT, USA; ^4^Department of Biomedical Engineering, Federal University of ABCSanto Andre, SP, Brazil; ^5^Department of Mechanical Engineering, Massachusetts Institute of TechnologyCambridge, MA, USA; ^6^Department of Brain and Cognitive Sciences, Massachusetts Institute of TechnologyCambridge, MA, USA

**Keywords:** discrete, rhythmic, internal models, primitives, arm movements

## Abstract

Given the vast complexity of human actions and interactions with objects, we proposed that control of sensorimotor behavior may utilize dynamic primitives. However, greater computational simplicity may come at the cost of reduced versatility. Evidence for primitives may be garnered by revealing such limitations. This study tested subjects performing a sequence of progressively faster discrete movements in order to “stress” the system. We hypothesized that the increasing pace would elicit a transition to rhythmic movements, assumed to be computationally and neurally more efficient. Abrupt transitions between the two types of movements would support the hypothesis that rhythmic and discrete movements are distinct primitives. Ten subjects performed planar point-to-point arm movements paced by a metronome: starting at 2 s, the metronome intervals decreased by 36 ms per cycle to 200 ms, stayed at 200 ms for several cycles, then increased by similar increments. Instructions emphasized to insert explicit stops between each movement with a duration that equaled the movement time. The experiment was performed with eyes open and closed, and with short and long metronome sounds, the latter explicitly specifying the dwell duration. Results showed that subjects matched instructed movement times but did not preserve the dwell times. Rather, they progressively reduced dwell time to zero, transitioning to continuous rhythmic movements before movement times reached their minimum. The acceleration profiles showed an abrupt change between discrete and rhythmic profiles. The loss of dwell time occurred earlier with long auditory specification, when subjects also showed evidence of predictive control. While evidence for hysteresis was weak, taken together, the results clearly indicated a transition between discrete and rhythmic movements, supporting the proposal that representation is based on primitives rather than on veridical internal models.

## Introduction

E pur si muove (and yet it moves).

Galileo Galilei, June 22, 1633

One core question in motor neuroscience is how neural control interacts with the peripheral mechanics of the body: What aspects of skilled movements are controlled by the nervous system and what aspects are attributable to intrinsic limb mechanics? What does the central nervous system “know” about the dynamics of its body? Neural control of movements necessitates some internal knowledge of the limb dynamics to predict and integrate the consequences of its commands to the mechanical periphery. However, it appears unlikely that the brain can make real-time predictions based on a veridical model of detailed body dynamics, especially if that model must include the dynamics of objects to be manipulated. Neural information transmission is extremely slow, which leads to substantial communication delays. This is particularly important for fast movements, where there is no time to correct for errors, yet complicated non-linear mechanical effects such as Coriolis accelerations become prominent. Consider wielding a whip: using a veridical model to predict the mechanics of this flexible body interacting with a compressible gas (air) would tax even modern supercomputers. Using this model to find an “optimal” action to place the end of the whip at a desired location in space is vastly more challenging. Yet, humans solve this apparently intractable problem and can manipulate a whip with astonishing skill. But how? Observing the solution that humans adopt is revealing: it appears to consist of essentially two relatively simple movements, a large sweeping arm motion combined with an extremely fast (and precisely-timed) wrist “flick.”

We propose that the nervous system generates complex actions by combining elements from a limited set of modules or primitives. In recent work (Hogan and Sternad, 2012, 2013) have outlined a theoretical framework proposing dynamic primitives for the control of sensorimotor behavior. Specifically, we argued that actions are composed of submovements, oscillations, and mechanical impedances, the latter to account for interaction with external objects. Note that these primitives are dynamic attractors giving rise to the observable movements. Submovements and oscillations in particular generate the observable discrete and rhythmic movements, respectively. This work extends previous experimental and modeling work by Sternad and colleagues that examined complex movements as a combination of discrete and rhythmic elements (Sternad et al., 2000; Sternad, 2008). Generating actions on the basis of primitives may afford more computational efficiency but, in turn, may also imply less versatility. The present experimental study probes into such limitations to test whether rhythmic and discrete movements indeed reflect distinct classes of behavior, i.e., primitives.

A few studies have provided support that discrete and rhythmic movements are mediated by different neural circuits. An fMRI study revealed significantly different cerebral activation for the two types of movements (Schaal et al., 2004). In continuous rhythmic wrist movements cerebral activation was largely confined to unilateral primary motor areas, whereas a sequence of discrete movements elicited strong additional activity in the bilateral parietal cortex and cerebellum. Subsequent behavioral results reinforced this difference. For example, Ikegami et al. (2010) showed that adaptation to altered visuomotor conditions was almost fully transferred from discrete to rhythmic performance, while there was minimal transfer in the reverse direction. Howard et al. (2011) reported that when learning reaching movements in force fields with different directions, interference between reaching in different force fields was reduced when each field was performed in either a rhythmic or discrete manner.

While these studies present intriguing differences between rhythmic and discrete performance, a stronger test for the existence of primitives would be to reveal limitations that arise when the two movements coexist. Previous experimental work by Sternad and colleagues examined movements that combined rhythmic and discrete elements in uni- and bimanual, single- and multi-joint tasks. Probing superposition of rhythmic and discrete movements at random phasing revealed that discrete displacements preferentially occurred in limited phase windows of the ongoing rhythmic movements (Sternad et al., 2000, 2002; Sternad and Dean, 2003; Wei et al., 2003). If control were based on a veridical internal model, it should be possible to superimpose or merge discrete and rhythmic movements in any task-specified way, subject only to the limitations of the musculo-skeletal system. Other evidence comes from slow movements, where the slow response of the musculo-skeletal system becomes less important. If control was based on a veridical internal model, it should be possible to perform rhythmic movements arbitrarily slowly. However, several studies indicated that repetitive movements, if performed sufficiently slowly, transition to a sequence of discrete movements (Doeringer and Hogan, 1995; Adam and Paas, 1996; Hogan et al., 1999; van der Wel et al., 2010). Further, evidence from stroke patients showed that their earliest recovered movements are “quantized,” but become smoother and more continuous with recovery (Krebs et al., 1999; Rohrer et al., 2002, 2004).

The present study adopted another approach to expose limitations arising from a modular representation. Based on the reasonable assumption that fast movements have high computational and mechanical demands (more prominent velocity- and acceleration-dependent forces and time-stressed planning to compensate for them), we stressed the neuromechanical system by driving it fast. Performing a sequence of discrete point-to-point movements at an increasing frequency should challenge the central nervous system, leading to an eventual “break-down” of movement performance, either due to mechanical or computational limitations. We hypothesized that if the CNS operates on the basis of primitives, discrete movements with precisely timed starts and stops would become computationally more challenging with speed and give way to rhythmic movements as an easier way to satisfy the timing demands. Importantly, this transition should occur after the neuromuscular system had reached its mechanical limits and, ideally, within the course of a single cycle (*Hypothesis 1*).

In systems that have multiple stable states, transitions between them typically depend not only on the present state, but also on the history of states. Therefore, transitions in opposite directions may exhibit an asymmetry, usually termed hysteresis. This is particularly the case in systems that have a lag between input and output, as has been demonstrated in numerous physical systems. Importantly, this type of hysteresis has also been identified in biological systems and, specifically, in perceptual-motor systems. For example, the perception of motion direction shows hysteresis (Williams et al., 1986; Hock et al., 2005). In human locomotion the transition from walking to running typically happens at a speed greater than in the reverse direction (Thorstensson and Robertson, 1987; Hreljac, 1995; Li, 2000). We therefore hypothesized that if discrete and rhythmic movements are generated by dynamic primitives with attractor stability, then transitions between these two movements should exhibit hysteresis (*Hypothesis 2*).

To test these two hypotheses we required subjects to perform a sequence of precisely timed discrete movements in synchrony with a decreasing and, subsequently, increasing metronome interval. We used a chirp signal where, after initially constant intervals, each successive interval first decreased by a fixed amount per cycle, then was constant for several cycles, then increased again by a fixed amount per cycle, and finally remained constant. Discrete movements require explicit demarcation by a non-zero interval of dwell time (Hogan and Sternad, 2007). To emphasize starting and stopping as a task criterion, the instruction explicitly specified this dwell time to be half of the movement time. To make this instruction even more explicit, we presented not only a sequence of short auditory beeps, but also prolonged auditory stimuli that exactly specified the required dwell duration. In this way, any deviation from the instructed dwell time could not be attributed to ambiguous instruction. If movement control is based on primitives, we hypothesized that transitions from discrete to rhythmic movements would occur, even if explicit temporal information about dwell time was available (*Hypothesis 3*).

A large body of research has examined control and adaptation of discrete reaching movements. The majority of these studies included visual control or examined visually evoked adaptations explicitly (Krakauer et al., 2000; Shabbott and Sainburg, 2009, 2010). As visual information typically predominates over proprioceptive and auditory information, visual information and visually-based error-correction processes may mask aspects of representation and may potentially bias neural or computational constraints due to primitive-based representation. To avoid such masking or biasing effects, the experiment tested performance with and without visual information. We hypothesized that the transition from discrete to rhythmic movements would occur for both visual conditions, but may be facilitated under no-vision conditions (*Hypothesis 4*).

## Methods

### Participants

Ten volunteers participated in this experiment (31 ± 12 years old, 6 male and 4 female). Nine subjects were right-handed according to the Edinburgh handedness test, one subject was left-handed. Prior to data collection, the participants were informed about the experimental procedure and signed an informed consent form approved by MIT's Institutional Review Board.

### Experimental apparatus and data collection

**Figure 1:**
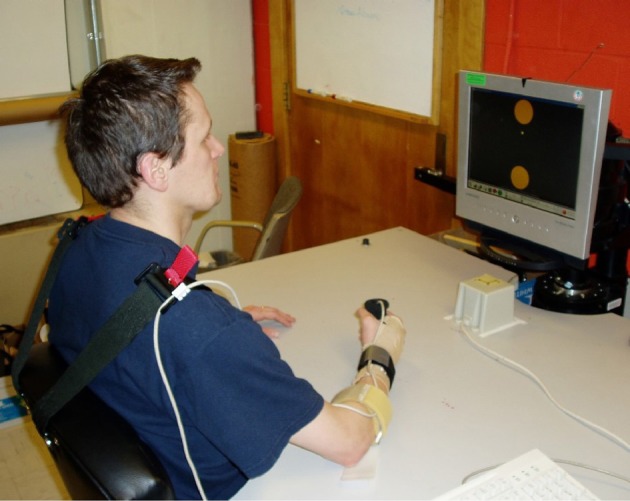
**Experimental set-up.** Subject holds a handle with a magnetic sensor attached that measures and shows its position online on the monitor in front. Subject is instructed to perform discrete movements forward (away from his body) and backward (toward his body), sliding on the horizontal table surface. Movement amplitude is indicated by two large circles on the monitor.

The participant was seated in front of a table, with the sternum close to the table edge (Figure 1). To fixate the shoulder position, two belts tied the upper body to the back of the chair. A mark on the table was used to locate each subject in a comparable position. The marked spot was in the subject's mid-sagittal plane, 23 cm away from the edge of the table (in the anterior-posterior direction) and ~26 cm distant from the subject's sternum. The height of the chair was adjusted to position the subject's upper arm to be ~45° to horizontal; the forearm rested on the table. From this neutral position, the subject could perform a reaching movement forward and backward in the sagittal direction, involving both shoulder and elbow joints without reaching the limits of their workspace. The forearm was mounted on a low-friction skid that reduced the static and kinetic friction on the surface during the movements. A brace stabilized the wrist to discourage wrist joint rotation.

Two circular targets were shown on a screen in a vertical arrangement to signal the amplitude of the movements (Figure 1). The two targets were at a distance of 14 cm from the neutral position, specifying a movement amplitude of 28 cm. The targets had a radius of 5 cm, which was relatively large so that accuracy requirements were minimal. The monitor was placed ~65 cm in front of the subjects to display the targets and a cursor showing their movements. The display gain was 0.5, showing the targets and movement amplitude at half of their real size. The subjects were asked to move from target to target by moving the handle back and forth on the table in the sagittal direction. A computer-generated metronome signal prescribed the timing for the movements.

The subject grasped a handle onto which a magnetic Flock of Birds sensor was attached (Ascension Technologies, Burlington, VT). The static accuracy and resolution were 0.25 and 0.08 cm, respectively. The combined weight of the sensor and handle was ~70 g, which is about 1/8 of the mass of the hand. The sampling frequency was 100 Hz. This frequency was sufficient as the frequency content of the motion was significantly below 50 Hz and anti-aliasing was not required. The position was zeroed with the handle in a neutral position shown by a mark on the tabletop. Data collection was controlled by a custom-made software routine written in Tcl on a computer running Linux Ubuntu operating system.

### Experimental conditions and procedure

**Figure 2:**
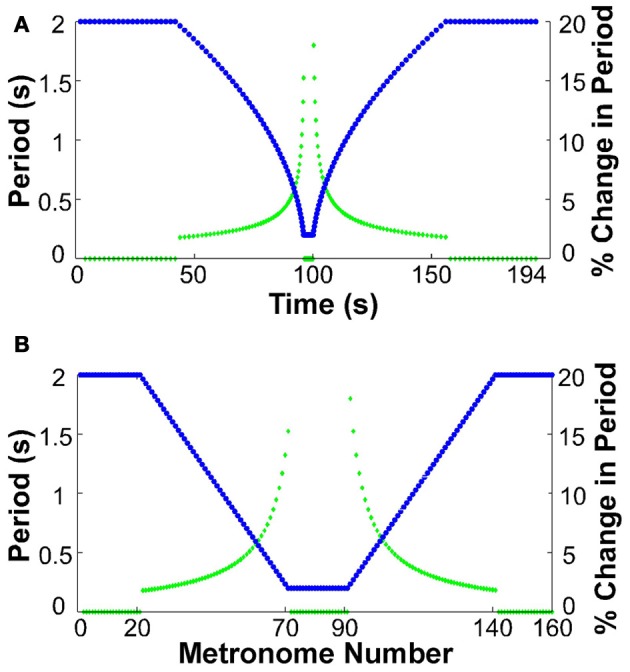
**Sequence of metronome intervals and their change in percent. (A)** Intervals (blue) and their corresponding percent change (green) displayed as a function of real time. **(B)** Intervals (blue) and their corresponding percent change (green) as a function of metronome number.

At the beginning of each trial participants placed their hand in the neutral position. All participants used their dominant hand. They were then instructed to move between targets in synchrony with the metronome sounds. The metronome sequence took the following form for all trials: the trial began with 20 sounds separated by an interval of 2 s, presenting a constant-period signal for 40 s. Subsequently, 50 sounds were produced where each interval decreased by 36 ms, ending at an interval of 200 ms. This short period was sustained for 20 sounds, equivalent to a duration of 4 s. After this constant-period interval, another 50 sounds with an increasing interval of 36 ms followed. The trial ended with 20 sounds of 2 s duration. The total trial duration was 194 s for a sequence of 160 moves. Figure 2 shows the sequence of periods as a function of time and also as a function of the number of metronome sounds. The figure also includes the change of inter-sound-interval as a percent of the previous inter-sound-interval to highlight that the changes were initially small, below 2%, but then grew to a maximum of 18%.

Subjects were instructed to perform point-to-point movements with an explicit dwell time to separate the movements into discrete movements. This dwell time should last for 50% of the metronome interval as accurately as possible. Subjects were instructed to maintain this discretization of movements for as long as possible, even when the pace increased and made the task more difficult.

This same timing sequence was presented under three different perceptual conditions. In the “vision” condition *V-short*, subjects had their eyes open and executed their movements to the displayed targets on the monitor. All metronome sounds had a duration of 50 ms, a short “beep.” In the “no-vision” condition, *NV-short*, subjects were asked to close their eyes, which removed the amplitude specification; the metronome sounds were still short with a 50 ms duration. In a third condition, *NV-long*, the sound duration was longer and adjusted at each interval to last 50% of the period, giving exact auditory information about the instructed dwell duration. Subjects again kept their eyes closed to encourage focus on the auditory timing information. These three conditions were repeated twice, both times in the same sequence. Comparison of the two trials allowed a test whether the performance features changed with practice. The total duration of these six experimental trials was ~25 min. Prior to data collection, each participant performed several moves with the metronome to familiarize him/herself with the task.

### Data reduction and analysis

**Figure 3:**
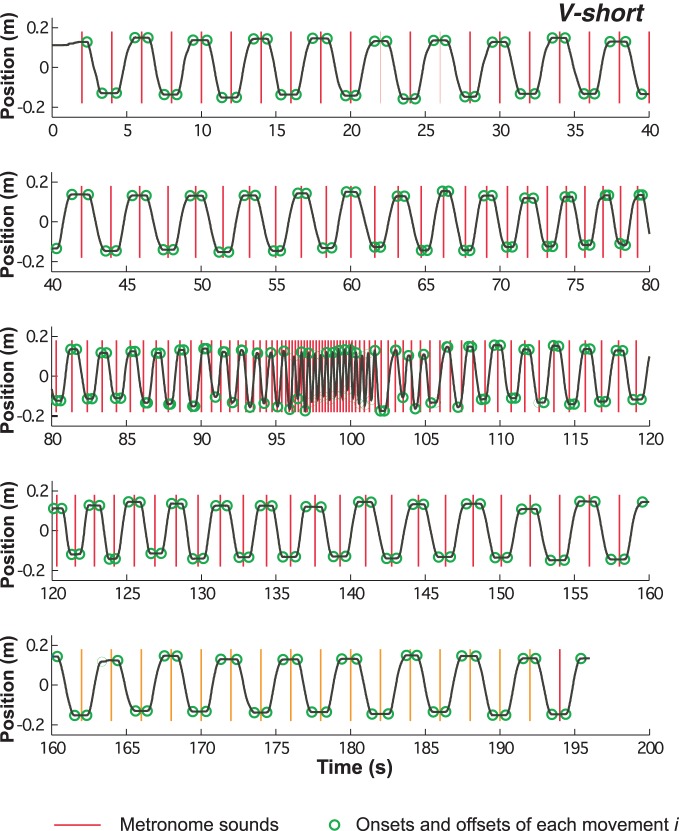
**Time series of continuous position over the entire trial of 194 s duration.** Red vertical lines indicate the onset of the metronome sounds, the green circles mark the onset and offset of each movement (see methods).

Of the 3D signals from the Flock-of-Birds sensor only displacements in the sagittal direction were processed. Figure 3 shows a complete time-series of one trial (condition *V-short*), divided into five segments due to the length of the trial. The time-series reveals the dwell times at the extreme positions in the initial and final slow-paced sections of the trial. As the pace increased, the dwell times steadily decreased and then disappeared. The dwell times reappeared with increasing metronome interval. The vertical lines denote the onset of the metronome sound; the circles mark the onset and offset of each movement.

To extract the changes of kinematics with the changing metronome pace and to identify whether a transition in control occurred, the kinematic signals were analyzed as follows. Before extracting quantitative markers, the position data were smoothed using a five-sample moving average filter, with centered filtering that did not introduce a lag (*smooth* function in Matlab®). Velocity was obtained numerically from the two-sample difference of the position signal, and was smoothed again with the same five-sample moving average filter. The acceleration signal was obtained by spline fitting and differentiation of the position signal (see below).

### Parsing into single movements

**Figure 4:**
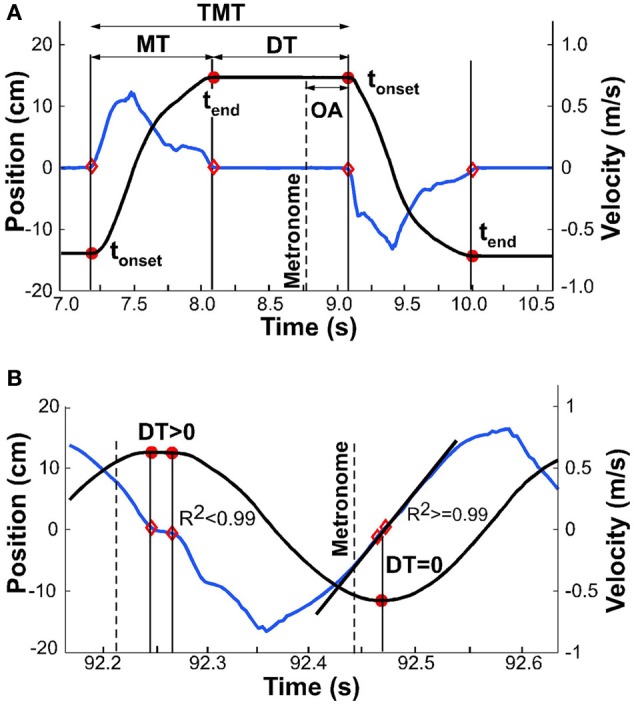
**Exemplary kinematic profiles to illustrate details of data analysis.** The black line denotes position, the blue line denotes velocity. **(A)** Movement time, dwell time, and total movement time are demarcated by onset and offset times, shown by red dots. Onset asynchrony is the temporal difference between metronome onset and movement onset. **(B)** Exemplary profile where detected onset and offset are in close proximity and dwell time is greater than zero (first segment of profile); onset and offset are identical (second segment) and dwell time is zero. Linear regression at this point yields *R*^2^ greater than 0.99.

For all analyses, the continuous kinematic data were parsed into single movements, delimited by *t*_onset_ and *t*_end_ (Figure 4A). Both onset and end of a movement were defined by the time when velocity crossed a threshold, defined as 3% of the peak velocity of the same movement. Given that all subsequent measures depended on this temporal demarcation, alternative thresholds of 1 and 5% were compared for their influence on subsequent analyses. As no significant differences were identified, we used 3% as the threshold for all subsequent analyses. Another option was to apply a fixed threshold, for example based on a threshold velocity. However, with decreasing interval and increasing velocity, a fixed threshold would favor earlier parsing. As this might have biased the calculations of dwell times (see below) in favor of our hypothesis, we chose the percent-based criterion.

This parsing analysis, however, faced a challenge when the movements merged to become approximately sinusoidal: the velocity decreased to zero but there was no longer any dwell time (Figure 4B). To eliminate false detections, a linear fit was applied to the velocity samples that were below the 3% threshold; the number of samples for this regression varied between 3 for fast movements up to 100 for slow movements (depending on the individual). *t*_onset_ and *t*_end_ were considered coincident when the *R*^2^ of the linear fit was above 0.99. The sample with the lowest velocity defined the time separating adjacent movements. To test the robustness of the onset and end times, we also ran the algorithm with an *R*^2^ cut-off of 0.95. The small differences that resulted did not have any effect on the subsequent analyses, most notably on onset and offset of dwell time.

### Movement time, dwell time, and total movement time

Movement time *MT*_*i*_, dwell time *DT*_*i*_, and total movement time *TMT*_*i*_ were defined as

$$\begin{array}{l} \;MT_{i}=t_{\text{end},i}-t_{\text{onset},i}\\ \;DT_{i}=t_{\text{onset},i+1}-t_{\text{end},i}\\ TMT_{i}=t_{\text{onset},i+1}-t_{\text{onset},i} \end{array}$$

where *i* and *i* + 1 denote the movement index. The duty cycle *DC*_*i*_ for each movement was defined as:

$$DC_{i}=MT_{i}/\text{​}\left(MT_{i}+DT_{i}\right)$$

By instruction, *DC*_*i*_ should be equal to 0.5; it became 1.0 when dwell time became zero.

### Transition between discrete and rhythmic movements

**Figure 5:**
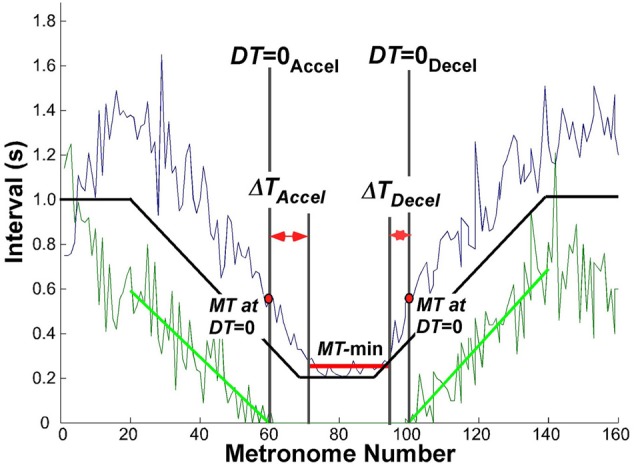
**Exemplary trial of one subject to illustrate the landmark events that were used to evaluate the transition from discrete to rhythmic movements.** The black solid line represents the instructed movement time; the green data show the dwell time, the blue data denote the movement time. The green line is the linear regression of dwell time between movement number 20 (end of initial steady state interval) and *DT* = 0_Accel_; in the decelerating portion between *DT* = 0_Decel_ and movement number 140 (start of steady state interval).

The criterion used to define a transition between discrete and continuous rhythmic movements was when dwell time became zero. In the accelerating portion of the trial, this time *DT* = 0_Accel_ was defined by the movement *i* that had zero *DT* and was followed by at least two more movements with zero *DT*. Similarly, for the decelerating portion, where rhythmic movements transitioned to discrete movements, *DT* = 0_Decel_ was defined as the last movement *i* with zero *DT* preceded by at least two movements with zero *DT*. This criterion proved to be very robust, as the onset and offset times remained unaffected by small changes in the parsing algorithm (see above). Figure 5 shows an exemplary trial with *MT* and *DT* displayed as a function of metronome number. For statistical comparisons we examined the movement times *MT* and metronome numbers associated with *DT* = 0.

To evaluate the hypothesized transition from discrete to continuous rhythmic movements, it was also necessary to determine the minimum movement duration that subjects could perform, which was typically longer than the shortest metronome interval. Inspection of the data showed that the region of minimum movement duration was not coincident with the trial segment of the 200 ms metronome intervals. Hence, to obtain a robust estimate of this interval when subjects reached and left their minimum movement duration, a window was defined using the following threshold (Figure 5):

$$MT\leq MT_{\min}+0.05\times (MT_{\max}-MT_{\min})$$

where *MT*_min_ and *MT*_max_ was the absolute minimum and maximum in the same trial, respectively. Successive *MT*s below this threshold defined the window length, which was typically shorter than the 20 metronome sounds that defined the constant-period interval. This window is shown in Figure 5 as a horizontal red line in the center of the trial. The mean *MT* in this interval was used as a robust estimate of *MT*-min.

These defined landmarks served to quantify the relation between *DT* = 0 and when minimum movement duration was reached. In particular, we defined Δ*T*_Accel_ as the interval between *DT* = 0_Accel_ and the start of *MT*-min, and Δ*T*_Decel_ as the interval between the end of *MT*-min and *DT* = 0_Decel_.

$$\begin{array}{l} \Delta \text{​}T_{\text{Accel}}=\text{start}(MT\text{-}\min)-DT=0_{\text{Accel}}\\ \Delta \text{​}T_{\text{Decel}}=DT=0_{\text{Decel}}-\text{end}(MT\text{-}\min) \end{array}$$

### Discreteness index

**Figure 6:**
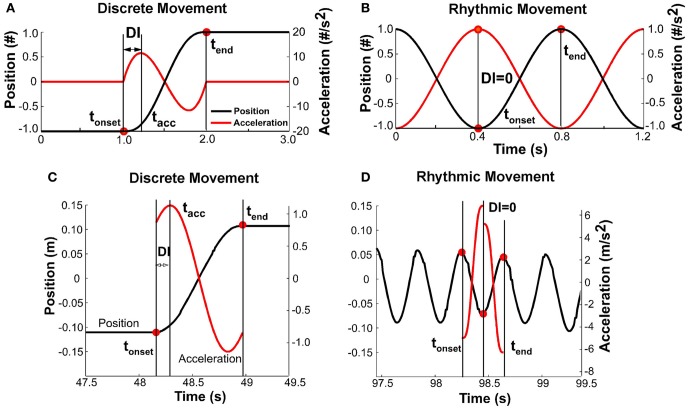
**(A)** Simulated profile of a discrete movement to illustrate the definition of the discreteness index, *DI*: the interval between movement onset and time of first peak acceleration divided by the total movement time. **(B)** Simulated profile of a rhythmic movement; the discreteness index is zero as the interval between movement onset and peak acceleration is zero. **(C)** Data profile of a slow movement where the position signal was approximated by a quintic spline and the acceleration profile was the double-differentiated signal. The movement has clearly demarcated dwell times and is discrete. **(D)** Data profile for a fast movement that was determined to be continuously rhythmic.

It was expected that with changing pace, the shape of the kinematic profile of each movement would change. To capture this modulation, a discreteness index *DI* was defined for each movement *i* based on the position and acceleration profiles (Figure 6). The *DI* was defined as the relative timing of the first peak in the acceleration profile *t*_acc_ with respect to the movement time:

$$DI_{i}=\left(t_{\text{acc},i}-t_{\text{onset},i}\right)\text{​}/MT_{i}$$

Figures 6A,B illustrate this measure for discrete and rhythmic movements, using simulated profiles for clarity. A discrete movement *DI* had a non-zero value, while for the sinusoidal movement, *DI* was equal to zero. For reference, the *DI* for a cycloidal movement is 0.25. A movement that minimizes jerk has a *DI* of about 0.20 (Hogan, 1984). Figures 6C,D shows data. As acceleration is notoriously noisy, the position signal of each movement (in the interval between onset and offset, not considering dwell time) was approximated via a quintic spline. The second derivative of the spline served as the acceleration profile needed for the calculation of the discreteness index. As the figure for the fast rhythmic movement shows, the quintic spline was different for the first and second half of the movement. Importantly, though, the DI was zero.

### Onset asynchrony

To quantify how movements were synchronized with the metronome sounds, a measure of onset asynchrony was defined as the temporal difference between each movement's onset and the corresponding metronome sound's onset (Figure 4A):

$$OA_{i}=t_{\text{onset},i}-t_{\text{metronome},i}$$

Positive numbers indicated that the movement onset lagged the onset of the metronome sound. Determining this difference needed care as in the faster portions of the trial subjects occasionally skipped cycles. To avoid erroneous matching yielding onset asynchronies longer than a cycle, the algorithm matched the metronome and movement times with increasing time for the first accelerating part of the trial; for the second decelerating part of the trial, the algorithm started at the end of the trial and worked backwards to determine correspondence between metronome and movement times.

### Statistical analyses

Comparison of the different measures in the three conditions was performed using two-way analysis of variance, with condition and trial as fixed factors and subjects as random factor. If variables of the accelerating and decelerating portion were compared, a three-way 3 (condition) × 2 (Accel, Decel) × 2 (trial) ANOVA was conducted. If different segments of the same trial were compared, a 3 (condition) × 2 (segment) × 2 (trial) ANOVA was used. Student *t*-tests were used to compare time estimates with metronome times. The significance level was always set at α = 0.05.

## Results

An exemplary time series of one subject in condition *V-short* is shown in Figure 3. The figure shows that in the first and last portion of the trial dwell times at the position extrema demarcating discrete movements were pronounced, but these plateaus decreased and eventually merged at ~90 s into the trial. They reappeared at approximately at 115 s in the trial. Movements appeared to be synchronized with the metronome, although strict synchronization between metronome sounds and movement peaks was lost during the short interval section in the center of the trial. In fact, almost all subjects skipped or even inserted cycles in the fast portion of the trial. In 22 out of 60 trials the number of cycles did not correspond to the metronome-specified number. However, the occurrence did not show any systematic dependency on the task condition. Supported by spontaneous comments of the subjects following the experiment, the fast movements were very difficult to perform and synchronization with the metronome could no longer be sustained.

Movement amplitudes were relatively invariant in the slower portions of the trial following the visual targets but became more variable and smaller when the movements became fast. As is to be expected, the decrease in amplitude in the short-interval section was more significant in the no-vision conditions. This corroborated that maintaining synchrony with the metronome was indeed taxing as intended and subjects compromised amplitude to facilitate the increasingly faster timing.

### Synchronization with metronome

**Figure 7:**
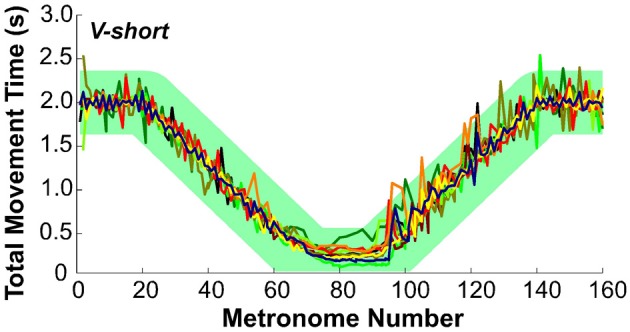
**Total movement time of all 10 subjects across trial 1 in condition *V-short*.** The green band illustrates the instructed movement time with an envelope of ±350 ms that includes 99% of all data.

To evaluate whether subjects followed the task instructions and produced the movement times as specified, Figure 7 presents the total movement time, *TMT*, defined as the temporal difference between two consecutive movement onsets, plotted for each movement and for all subjects in condition *V-short*. The green band is an envelope around the instructed intervals with a width of ±350 ms that encompassed 99% of all values. The results are displayed as a function of metronome number. This convention avoided inconsistent plotting across individuals when movement cycles were skipped, which happened frequently during the short-interval section of the trial.

To test the subjects' synchronization to the instructed time, *TMT* was regressed against the metronome interval. For perfect synchronization, the slope of this regression should be the identity line. For all 10 subjects in all three conditions the mean slope was 0.98 ± 0.025 (one standard deviation), with *R*^2^-values ranging between 0.89 and 0.99. There was no significant difference between the three conditions. Hence, it can be concluded that overall, subjects maintained synchronization with the metronome.

### Discrete movements and dwell time

**Figure 8:**
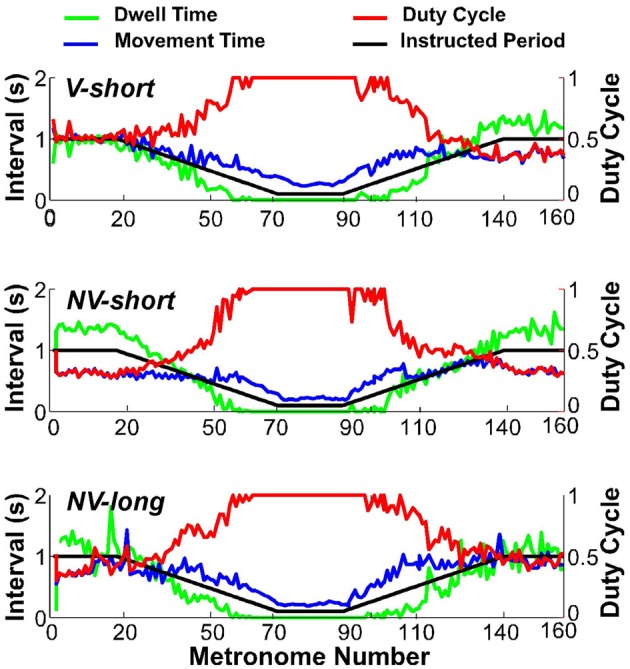
**Movement time (blue), dwell time (green), and duty cycle (red) of one representative subject in all three conditions.** The black line serves as reference denoting the instructed interval.

To assess whether subjects satisfied the task instructions and produced discrete movements separated by dwell times, the TMT was split into its component times, dwell time *DT*, and movement time *MT*. Figure 8 shows one subject's data in the three conditions (first trial), with *DT* in green, *MT* in blue, and the duty cycle *DC* in red; the black line represents the metronome-specified movement interval. As is clear from the duty cycle, the instructed dwell time was not always achieved, neither in the steady state portions of the trial, nor in the accelerating and decelerating portions. This subject did not display a duty cycle of 1.0 in the beginning of conditions *NV-short* and *NV-long*. Other subjects showed similar deviations in this part of the trial but no systematic pattern or dependency on condition could be identified.

**Figure 9:**
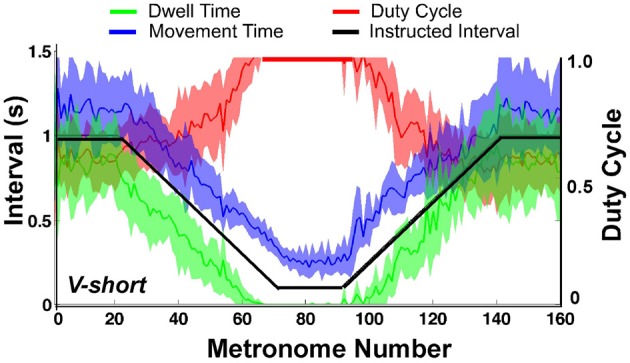
**Movement time (blue), dwell time (green), and duty cycle (red) averaged over all subjects.** The black line represents the instructed interval. The shaded areas around movement time, dwell time, and duty cycle denote ± one standard deviation around the mean of 10 subjects.

When the movement intervals changed after movement number 20, the duty cycle was not maintained for long. Following a steady decrease, dwell time *DT* vanished at about metronome number 60 in the decelerating portion of the trial. In the accelerating portion of the trial, *DT* re-appeared approximately at metronome number 100, followed by a declining duty cycle. Figure 9 summarizes these observations for the condition *V-short*. The figure shows the average of all 10 subjects; the shaded error bands represent ± one standard deviation across subjects.

The important observation in Figures 8, 9 is that dwell times vanished before movement durations had reached their minimum in the center portion of the trial; in the accelerating portion of the trial, dwell times re-appeared after movement time had already increased. According to the task instructions, dwell times should have declined continuously proportional to movement duration until minimum movement time was reached. In contrast, *Hypothesis 1* predicted that discrete movements would be abandoned early in favor of computationally less demanding rhythmic movements.

### Minimum movement time

To test *Hypothesis 1*, we first determined minimum movement time, *MT*-min, as reached in the center of the trial (see Figure 5 and methods). All subjects' values in the three perceptual conditions were submitted to a 3 (condition) × 2 (trial) ANOVA. Results revealed that the condition *V-short* showed a significantly longer *MT*-min of 248 ms, compared to 220 and 217 ms in the no-vision conditions *NV-short* and *NV-long*, *F*_(2, 47)_ = 5.76, *p* < 0.001. *Post-hoc* tests showed that the 248 ms in the *V-short* condition was significantly different from the other two conditions, which did not differ from each other (*p* < 0.01). The two trials showed no significant difference. Pair-wise student *t*-tests compared *MT*-min against the instructed movement interval of 200 ms and detected a significant difference for all subjects in all three conditions, *t*_(59)_ = 4.82, *p* < 0.0001. This result again corroborated that, as intended, subjects had reached their limit of movement duration in all conditions. This conclusion was further supported by the fact that this effect was not influenced by practice. The shorter times in the no-vision conditions correlated with a decrease in amplitudes. As expected from *Hypothesis 4*, subjects complied better with timing demands of the task when visual information was removed.

### Movement time at transition to rhythmic movements

We next determined the times when dwell time disappeared and reappeared in the decelerating and accelerating portions and the corresponding movement times, *MT*_*DT* = 0_ (see Figure 5). Subjecting *MT*_*DT* = 0_ to a 3 (condition) × 2 (Accel-Decel) × 2 (trial) ANOVA revealed a significant difference between conditions, *F*_(2, 80)_ = 4.77; *p* < 0.01, while there was no difference between the accelerating and decelerating segments, nor a significant difference across the two trials. Subjects performed with an average movement time of 463 and 457 ms for *V-short* and *NV-short*, respectively; for *NV-long* the average movement time was 536 ms, which was significantly longer than the other two conditions, which did not differ from each other (*p* < 0.01). These times were significantly longer than the minimum movement times (*p* < 0.01). This result supported *Hypothesis 1.* However, the symmetry in movement times between the accelerating and decelerating portions was not consistent with *Hypothesis 2*. Importantly, these results also revealed that zero dwell times started earlier in the condition with the explicit temporal information. This finding is consistent with *Hypothesis 3* predicting that explicit temporal information will not prevent a transition. In fact, the result shows that the transition occurred even earlier in the short beep conditions.

### Hysteresis

Despite the symmetry in movement times at *DT* = 0, inspection of Figures 8, 9 shows that the first and second transition, i.e., start and end of the minimum movement time, *MT*-min, were slightly shifted relative to the instructed movement times or metronome indices, suggestive of hysteresis. To quantify this observation we defined the intervals between *DT* = 0 and *MT-min* in the accelerating and decelerating portions, Δ*T*_Accel_ and Δ*T*_Decel_, for each subject (see methods and Figure 5). A first set of *t*-tests confirmed again that these intervals were significantly different from zero for all subjects and conditions (*p* < 0.01). Subsequently, Δ*T*_Accel_ and Δ*T*_Decel_ were subjected to a 3 (condition) × 2 (Accel-Decel) × 2 (trial) ANOVA. Results showed a significant difference between the two transitions, *F*_(1, 76)_ = 24.77, *p* < 0.0001, but no differences between the three perceptual conditions, nor between the two trials. Dwell time vanished on average 11 movements before the minimum movement time was reached; in the decelerating portion movement time *MT* increased on average 7 movements before dwell time *DT* re-appeared. This asymmetry is consistent with a hysteresis effect as predicted by *Hypothesis 2*. The persistence of fast rhythmic movements after metronome intervals had increased lasted ~2.8 s, which is relatively long and makes a mechanical cause improbable.

### Discreteness index

**Figure 10:**
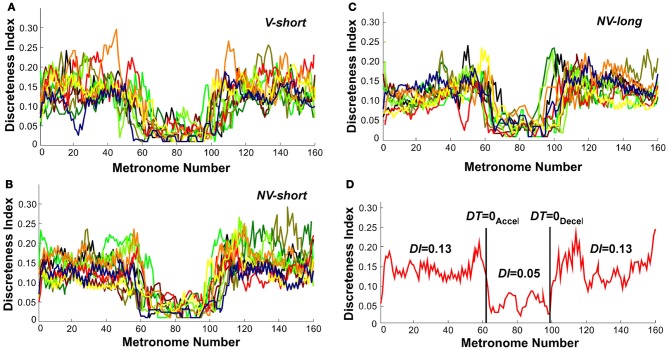
**Discreteness index values of all subjects across metronome number. (A–C)** All subjects' values in the three perceptual conditions. **(D)** A single subject's trial; the vertical lines show the trial segmentation delineated by the time when dwell time is zero. The discreteness indices *DI* calculated for the three segments are shown.

To further identify the hypothesized transition we examined changes in the continuous movement kinematics in terms of the discreteness index *DI*; this index quantified the shape in the bell-shaped velocity profile of each movement. Figures 10A–C shows the *DI* for all subjects in the three perceptual conditions. As can be seen, *DI* changed rapidly near movement number 60 and again at 100 in a similar fashion for all three conditions. Figure 10D shows an exemplary single subject's trial in the *V-short* condition to further illustrate the abrupt change in *DI*. For statistical analysis, the trial was parsed into three segments: before *DT* = 0_Accel_, the center portion, and after *DT* = 0_Decel_. This individual's *DI* values were 0.13 in first and last segments, with a drop to 0.05 in the middle segment. A 3 (segment) × 3 (condition) × 2 (trial) ANOVA on all subjects' data identified significant differences across segments, *F*_(2, 76)_ = 431.42, *p* < 0.0001. There was no main effect or interactions for the perceptual conditions and trials. The average *DI* across the three conditions was 0.13 before *DT* = 0_Accel_, 0.04 in the center portion, and 0.14 after *DT* = 0_Decel_, similar to the exemplary subject in Figure 10D. This measure captured changes in each trajectory and revealed a relatively abrupt transition from discrete to rhythmic movement control strategies, consistent with *Hypothesis 1*.

### Onset asynchrony

**Figure 11:**
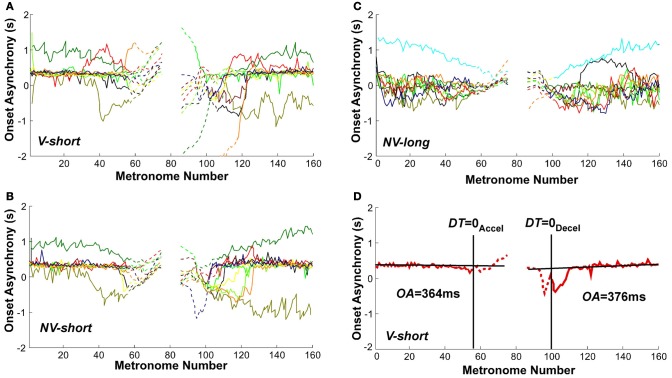
**Onset asynchrony values of all subjects across metronome number. (A–C)** All subjects' values in the three perceptual conditions. **(D)** A single subject's trial with line fits. The linear regressions regressions were performed over the intervals 0–40 and 120–160 of metronome numbers. The red lines are dashed after *DT* = 0_Accel_ and before *DT* = 0_Decel_.

Given that the task required synchronization with a metronome, the synchrony of movement onset with the auditory stimulus was analyzed to assess entrainment and predictive control. Figures 11A–C shows the sequence of onset asynchronies, the difference between movement and metronome onset. The data from all individual subjects in all three conditions showed that there was considerable variability across subjects. Despite this inter-individual variability, the majority of subjects exhibited a relatively constant onset asynchrony *OA* from the trial start until movement numbers 50–60, which lasted well beyond the initial long-interval section. Similarly, the second part of the trial showed relatively constant values of *OA* for the last 40–50 movements. The *OA* values after *DT* = 0_Accel_ and before *DT* = 0_Decel_ are shown as dashed lines. To highlight these observations, Figure 11D shows *OA* values in a single subject's trial in the *V-short* condition. The straight black lines were obtained from linear regression over the first 40 and last 40 *OA* estimates in each trial. As the slopes did not differ from zero in all conditions, we used the mean values to estimate representative onset asynchrony for the accelerating and decelerating segment and condition.

A 3 (condition) × 2 (segment) × 2 (trial) ANOVA identified a main effect for condition, *F*_(2, 106)_ = 6.755, *p* < 0.002, and a main effect for segment, *F*_(1, 91)_ = 5.97, *p* < 0.05. There were no other main effects or interactions. In the two conditions with the short metronome sound, *V-short* and *NV-short*, subjects showed an onset asynchrony of 536 and 399 ms; in *NV-long* it was only 129 ms, which was significantly different from the other two, as shown in *post-hoc* tests (*p* < 0.01). The delayed onset of the discrete movement in the two conditions with the short metronome sounds indicates reactive responses to the auditory stimulus, while the shorter onset asynchrony suggests some degree of predictive control. The long reaction times also suggest that the movements were complex and demanded substantial neural resources. The mean onset asynchronies in the first and second segment of the trials were 469 and 240 ms, respectively. Even though this difference between the two segments does not directly test hysteresis, it may still be consistent with the hysteresis effect shown above (*Hypothesis 2*). Summarizing, explicit auditory timing information apparently elicited predictive control.

## Discussion

This study attempted to test whether movement control is based on a small set of dynamic primitives, specifically submovements and oscillations that underlie observable discrete and rhythmic movements. We hypothesized that coordination based on primitives is computationally less demanding. However, representation in the form of dynamic primitives may also limit the versatility of coordinated movements that can be performed. One such limitation may be the speed with which a sequence of discrete movements can be executed. If rhythmic primitives are less demanding of neural resources then, when stressed by requiring rapid action, the system may merge a sequence of discrete movements into a continuous rhythmic movement.

### Transitions are not due to limitations of the motor periphery

The findings unambiguously demonstrated a switch from the instructed discrete movements to continuous rhythmic performance as TMT was reduced and another switch back to discrete movements as TMT increased again. This could not be attributed to limitations of the peripheral neuro-musculo-skeletal system. Despite the fact that subjects matched their TMTs to the metronome interval, they did not preserve the instructed dwell times, but progressively reduced them. The movement times at which dwell times disappeared were significantly longer than the minimum movement time achieved.

This alone might be attributed to a limited peripheral response speed or bandwidth, because, for a given metronome interval, a movement with dwell time has higher frequency components than a movement without dwell. Stated in the time domain, a movement with infinitesimal dwell time has larger accelerations than a smoothly rhythmic movement (Hogan and Sternad, 2007). However, changing the sensory condition significantly affected the movement duration at which dwell times disappeared—536 ms for *NV-long* vs. 463 and 457 ms for *V-short* and *NV-short*, respectively. In the *NV-long* condition, the passage to zero dwell time cannot be attributed to limited peripheral response speed, because subjects were demonstrably capable of faster movements with non-zero dwell times.

In addition, in all subjects and conditions, the discreteness index showed an abrupt change around metronome number 60 and 100. It dropped rapidly to zero approximately coincident with dwell time reaching zero as TMT decreased; it recovered rapidly from zero approximately coincident with dwell time increasing from zero as TMT increased again. Both of these observations are as predicted by *Hypothesis 1*. Discrete movements with precisely timed starts and stops require more neural resources than smoothly rhythmic movements (Schaal et al., 2004). As available movement time decreased, the increasing demand on neural resources evoked a switch to the neurally simpler rhythmic movements. Once available movement time increased again, the more challenging discrete movements were reinstated.

### Weak signs of hysteresis

A common phenomenon in systems with more than one stable state is that transitions between these stable states may display hysteresis: transitions in one direction occur at different parameter values than in the reverse direction. To test *Hypothesis 2*, the experiment included accelerating and decelerating portions that induced transitions from discrete to rhythmic movements and back from rhythmic to discrete movements, respectively. However, the TMT when dwell time reached zero as metronome intervals decreased was not significantly different from the TMT when dwell time increased from zero as metronome intervals increased again. This result is not consistent with *Hypothesis 2*.

Nevertheless, some asymmetry in the transitions was evident. The fastest (rhythmic) movements persisted for several seconds (2.8 s on average) after the metronome intervals began to increase again. This was followed by a faster rate of increase of movement time and dwell time with metronome number than their rate of decrease during the accelerating portion of the trial. The exact origin of this phenomenon requires further investigation.

### Explicit auditory information promoted rhythmic movements

One condition was included that presented explicit auditory information about the dwell duration in order to test whether this would guide subjects to produce the required dwell time. *Hypothesis 3* stated that explicit auditory information about dwell duration should not be able to prevent the transition between the two primitives: transitions should occur when the neural system was challenged and induced to resort to simpler solutions. Indeed, as predicted, auditory specification of dwell times did not facilitate the discrete movements and the weak hysteresis effect in the decelerating portion was unaffected by the different auditory conditions. However, there was one effect of prolonged auditory stimuli that was counter to expectations: when the metronome sounds were longer, subjects switched to rhythmic movements *earlier* and switched back to discrete movements *later* than in the short metronome conditions. This effect may be due to the fact that “filling” half of the time interval with sound made the auditory signal appear more periodic: sound and no-sound alternated periodically. The more rhythmic nature of the signal may have entrained the movement and induced rhythmic behavior.

The different auditory stimuli produced one additional effect. The two conditions with the short sound revealed a considerable delay between metronome to movement onset, 536 and 399 ms, that clearly indicated a reactive response. This is not surprising as the intervals of 2 s in the beginning and end of the trial were longer than humans can temporally integrate and perceive as rhythmic (Fraisse, 1984). In contrast, in the long-beep condition the delays from metronome onset to movement onset were considerably shorter (129 ms). In this condition the silent interval was at most 1 s, alternating with 1 s of sound. This sound pattern can easily be perceived as periodic. Human subjects readily predict periodic signals and have been shown to reduce the phase lag of their responses or even exhibit phase lead (Aschersleben, 2002). This distinctive feature of responses to periodic stimuli may also account for the transitions to and from rhythmic performance at longer metronome intervals. In effect, the auditory stimulus was more readily perceived as rhythmic and may have entrained a primitive rhythmic motor behavior (Mates et al., 1994). If so, this raises the intriguing possibility that *perception* as well as action may be based on dynamic primitives.

The substantially longer delays in the *V-short* and *NV-short* conditions suggest that subjects only reacted to the metronome trigger—without any prediction. Reactive behavior during the long inter-beep intervals of 2–1.5 s is not unexpected, although the reaction times were approximately 2–3 times longer than simple auditory reaction times. Moreover, the same reaction times persisted into much shorter intervals, even though the interval changes were systematic and predictable. These long reaction times may indicate the demands of movement planning (Henry and Rogers, 1960; Sternberg et al., 1978). It would appear that simple reaching movements with a timed dwell demanded considerable neural resources.

### Visual information has negligible influence on transitions

We speculated in *Hypothesis 4* that visual information may mask primitive-based transitions and performance with visual information excluded may better reveal transitions due to neural or computational constraints. However, none of the transition effects showed any dependency on the presence of visual information. One possible explanation for this lack of effect could be that in the no-vision conditions, where the spatial targets were not visible, subjects may have decreased their amplitudes to maintain discrete movements longer, which could have cancelled the hypothesized effect. The only effect that depended on vision was the minimum movement time reached. In the vision condition the minimum movement time was ~25 ms longer compared to the no-vision conditions. This difference was probably due to the fact that subject tried harder to maintain the target amplitude while when their eyes were closed they traded amplitude for timing accuracy.

## Summary and conclusions

The results are consistent with the overall hypothesis that control is based on primitives. Transitions from discrete to rhythmic movements occurred before the neuro-mechanical system had reached its maximum performance and the kinematic profiles changed rather abruptly. On the other hand, the evidence for hysteresis was weak. Interestingly, the transition from discrete to rhythmic was facilitated by long auditory metronome sounds, counter to expectations, indicating that perceptual rather than biomechanical factors influenced the transition. These results complement findings that revealed how continuous movements, if performed sufficiently slowly, decompose into chunks or submovements. Evidence for this intermittent control has been seen in healthy subjects and patients with lesions (Doeringer and Hogan, 1998; Krebs et al., 1999). In particular, slow rhythmic movements may transition to a sequence of discrete movements (Adam and Paas, 1996; van der Wel et al., 2010). Our experimental results add one more piece of evidence supporting the hypothesis that control is based on dynamic primitives.

### Conflict of interest statement

The authors declare that the research was conducted in the absence of any commercial or financial relationships that could be construed as a potential conflict of interest.
